# Iron depletion has different consequences on the growth and survival of *Toxoplasma gondii* strains

**DOI:** 10.1080/21505594.2024.2329566

**Published:** 2024-03-20

**Authors:** Eléa A. Renaud, Ambre J. M. Maupin, Yann Bordat, Arnault Graindorge, Laurence Berry, Sébastien Besteiro

**Affiliations:** LPHI, University Montpellier, Inserm, CNRS, Montpellier, France

**Keywords:** Acute toxoplasmosis, chronic toxoplasmosis, iron depletion, bradyzoites, cystogenic strains

## Abstract

*Toxoplasma gondii* is an obligate intracellular parasite responsible for a pathology called toxoplasmosis, which primarily affects immunocompromised individuals and developing foetuses. The parasite can scavenge essential nutrients from its host to support its growth and survival. Among them, iron is one of the most important elements needed to sustain basic cellular functions as it is involved in a number of key metabolic processes, including oxygen transport, redox balance, and electron transport. We evaluated the effects of an iron chelator on the development of several parasite strains and found that they differed in their ability to tolerate iron depletion. The growth of parasites usually associated with a model of acute toxoplasmosis was strongly affected by iron depletion, whereas cystogenic strains were less sensitive as they were able to convert into persisting developmental forms that are associated with the chronic form of the disease. Ultrastructural and biochemical characterization of the impact of iron depletion on parasites also highlighted striking changes in both their metabolism and that of the host, with a marked accumulation of lipid droplets and perturbation of lipid homoeostasis. Overall, our study demonstrates that although acute iron depletion has an important effect on the growth of *T. gondii*, it has a more profound impact on actively dividing parasites, whereas less metabolically active parasite forms may be able to avoid some of the most detrimental consequences.

## Introduction

The parasitic protist *Toxoplasma gondii* is responsible for a disease called toxoplasmosis that potentially affects humans and other warm-blooded vertebrates [1]. In immunocompetent individuals, the infection starts with an acute phase, caused by a highly multiplicative and invasive form called tachyzoite, and a long-lasting chronic phase involving an encysted slow-growing form called bradyzoite [2]. In immunodeficient individuals, brain-localized bradyzoites may reactivate, potentially leading to life-threatening encephalitis. Both developmental stages reside in specific intracellular compartments: tachyzoites actively invade their host cells and hijack lipids from the host plasma membrane to build a parasitophorous vacuole, whose membrane is an important interface supporting parasite survival and replication [3]. Upon the initiation of stage conversion into bradyzoites (which can be induced by a number of stresses), the parasitophorous membrane is heavily modified to build an underlying cyst wall containing various proteins and sugars, providing a protective barrier [4].

These obligate intracellular parasites have to acquire a number of vital nutrients from their host cells to support their growth and replication [5], as they have become auxotrophic for important metabolites, such as purines or cholesterol, which may be an interesting avenue for the design of novel antiparasitic strategies [6]. One of the key elements that developing parasites need to acquire is iron [7] which, through its involvement in protein cofactors such as haem or iron-sulphur clusters, is essential for many vital metabolic pathways in *T. gondii* tachyzoites [8,9]. Maintaining an appropriate iron balance becomes especially vital in the context of *T. gondii* infection [10,11] as the parasites need to acquire iron, while the host cells may deprive them of this essential metal. Consequently, host-driven iron withdrawal has the potential to inhibit the growth of these pathogens and can be a part of the interferon gamma-dependent response to *T. gondii* infection [12]. During the course of infection, the parasite may also naturally encounter tissues with variable iron availability. For example, the brain typically contains relatively low levels of iron compared to other organs because excessive iron in the brain can be harmful and is associated with certain neurodegenerative diseases [13].

Although there is only one *Toxoplasma* species, there are a variety of genotypes that differ in replication rate, ability to differentiate into bradyzoites, and virulence [14]. The lethality of strains for outbred laboratory mice is the phenotypic marker that was initially used to define the three archetypal clonal types (I, II, and III) of *T. gondii* [15]: type II and III genotypes are usually less virulent and more cystogenic than type I genotypes. These differences in laboratory mouse infections provide a suitable model for mimicking acute (with type I strains) and chronic (with type II and III strains) infections. In the context of human infections, type II strains are the most predominant among samples in America and Europe, followed by type I and type III (which are only occasionally found in humans) [16]. Wider sampling, both in terms of host and geographical distribution, has subsequently revealed a much more complex genetic diversity of the parasite [17]. The ability of strains to differentiate into bradyzoites is also a critical point in the efficacy of treatment, as tissue cysts are largely resistant to current drugs impacting tachyzoites.

In this study, we used the membrane-permeant intracellular Fe^2+^ chelator 2,2′-bipyridine (bipyridyl, BPD) to study the effects of iron deprivation on the survival of *T. gondii* parasites belonging to strains I, II, and III. The chelator had a marked impact on parasite growth and led to specific metabolic changes in both the host and the parasites, as illustrated by the accumulation of lipid droplets. However, we also noticed that iron depletion led to conversion into the bradyzoite stage for cystogenic parasites belonging to type II and III strains, which were consequently more likely to survive this metabolic stress.

## Materials and methods

### Parasites and cell culture

Tachyzoites of the RH [18] (type I), Prugniaud (PRU) [19,20] (type II) and NED [21] (type III) *T. gondii* reference strains were maintained by serial passage in human foreskin fibroblast (HFF, American Type Culture Collection, CRL 1634) cell monolayers or propagated in Vero cells (American Type Culture Collection, CCL 81), grown in Dulbecco’s modified Eagle medium (DMEM, Gibco) supplemented with 5% decomplemented foetal bovine serum, 2‐mM L‐glutamine and a cocktail of penicillin-streptomycin at 100 μg/ml.

### Plaque assays

Confluent monolayers of HFFs seeded in 24 well plates were infected with freshly egressed tachyzoites. Parasites were seeded onto host cells in the first lane of the plate at a multiplicity of infection of 0.03 parasite per host cell, and diluted to 1/4 in each subsequent row. The 2,2′-bipyridine (BPD) chelator (D216305, Sigma-Aldrich) was added 4 h post-invasion, and parasites were subsequently left to grow for 7–10 days for tachyzoites depending on the strain (tachyzoites from type II and III strains have a considerably longer doubling time than those from type I [14]). For iron supplementation experiments, FeCl_3_ was added at 100 µM simultaneously with the BPD as a source of Fe^3+^. Cells were then fixed with 4% v/v PFA and plaques were revealed by staining with 0.1% crystal violet solution (V5265, Sigma-Aldrich). Pictures of the plaques were acquired with an Olympus MVX10 microscope, and plaque areas were measured using ZEN software v2.5 (Zeiss). Plaque areas are expressed relative to the mean value of those generated by the respective control strain treated with the vehicle (Dimethyl sulphoxide -DMSO-, D5879, Sigma-Aldrich), which was set to 100%.

### IC_50_ determination

The IC_50_ values were determined from plaque assays performed with the following concentrations of BPD: 50, 25, 10, 5, 2.5, 1 and 0.5 µM. Plaque areas from three independent biological replicates were plotted relative to the compound concentrations using Prism 8 software (GraphPad).

### In vitro conversion to bradyzoites

We used alkaline pH-induced differentiation [22] as a control for the stage conversion. Briefly, monolayers of HFF grown on coverslips in 24-well plates were infected with 50,000 freshly egressed tachyzoites for 24 h in DMEM culture medium. The medium was then replaced with a differentiation medium made of Minimum Essential Medium (MEM) without NaHCO_3_ (Gibco), supplemented with 50 mM HEPES, 1% penicillin-streptomycin, 1% glutamine, and 3% FBS, and adjusted to pH 8.2. Parasites were then cultured at 37°C without CO_2_ and the medium was changed every two days during the entire duration of the experiment.

### Electron microscopy

HFFs were infected by the different *T. gondii* strains at a multiplicity of infection of 0.5 parasite per host cell (corresponding to ~ 10^5^ parasites) in Lab-Tek chamber slides (177437, Nunc). Infected cells were incubated for 40 hours with or without the chelator and were then chemically fixed for 2 h at room temperature using a fixation solution (2.5% glutaraldehyde in 0.1 M Cacodylate buffer, pH 7.4). The samples were stored at 4°C until further processing. Embedding in resin was carried out using a Pelco Biowave PRO+ Microwave processing system (Ted Pella). The details of the program are listed in Table S1. Samples were post-fixed with 1% osmium tetroxide in 0.1 M cacodylate buffer, pH 7.4. After washing, the samples were incubated in 2% uranyl acetate for 30 min at 37°C and further processed in a microwave oven. After washing, the samples were incubated in lead aspartate and pre-heated at 50°C. Dehydration was performed by increasing the acetonitrile concentration. The samples were then impregnated with EMbed-812 resin and polymerized for 48 h at 60°C. All chemicals were obtained from Electron Microscopy Sciences.

Thin serial sections were prepared using a UCT ultramicrotome (Leica) equipped with an ultra 35° diamond knife (Diatome). Section ribbons were collected on silicon wafers (Ted Pella) for SEM or on 100-mesh grids for TEM. Sections on wafers were imaged with a Zeiss Gemini 360 scanning electron microscope on the Montpellier ressources imagerie (MRI) EM4Bio platform under a high vacuum at 1.5 kV. Final images were acquired using a Sense BSD detector (Zeiss) at a working distance between 3.5 and 4 mm. Mosaics were acquired with a pixel size of 5 nm and dwell time of 3.2 µs. Sections placed on grids were imaged on a MET LaB6 JEOL 1400 Flash at 100 kV at the Electron Microscopy facility of the University of Montpellier (MEA).

### Immunofluorescence assay

Immunofluorescence assays (IFAs) of tachyzoites and differentiated bradyzoites were performed as described previously [23]. Briefly, monolayers of HFFs grown on coverslips were infected with tachyzoites, which were grown for the duration of the experiment, with or without the addition of BPD, and then fixed with 4% paraformaldehyde in PBS for 20 min at room temperature. Cells were washed three times with PBS, permeabilized with 0.3% Triton X-100/PBS for 15 min, and then saturated with 1% w/v bovine serum albumin (BSA)/PBS blocking solution for 30 min. Proteins were stained with primary antibodies for 1 h, followed by three washes with PBS, before incubation with secondary antibodies in a 1% BSA/PBS solution for 1 h. The primary antibodies used in this study and their respective dilutions were rabbit polyclonal anti-IMC3 [24] antibody diluted at 1/1,000, mouse monoclonal anti‐SAG1 [25] diluted at 1/1,000 (T3 1E5), mouse monoclonal anti-P21 [26] diluted at 1/200 (T8 4G10), mouse monoclonal anti-F1-ATPase beta subunit diluted at 1/1,000 (gift of P. Bradley), and rabbit polyclonal anti-pyruvate dehydrogenase E2 subunit [27] diluted to 1/500.

### Other fluorescent staining and microscopy-based quantifications

Cyst walls were stained with a 1/300 dilution of biotin-labelled *Dolichos biflorus* lectin (L-6533, Sigma-Aldrich) for 1 h and revealed using a 1/300 dilution of FITC-conjugated streptavidin (SNN1008, Invitrogen). DNA staining was performed on fixed cells by incubating them for 5 min in 1 μg/ml 4,6-diamidino-2-phenylindole (DAPI 62,248, Thermo Fisher) solution. All images were acquired at the MRI facility on a Zeiss Axio Imager Z2 epifluorescence microscope and analysed using ZEN v3.6 (Zeiss) and FIJI v1.53t (US National Institutes of Health) software. Nile red (72485, Sigma-Aldrich) staining was performed after the fixation and permeabilization steps and prior to antibody or lectin staining: the cells were incubated for 45 min with Nile red at a final concentration of 1 µg/mL. The area of lectin-stained cysts or Nile red-stained lipid droplets was measured using the contour (spline) tool of the ZEN software (Zeiss) after proper scale calibration.

### Lipidomic analysis

The parasite extracts for lipidomic analysis were prepared as follows. Vero cells were grown in 175 cm^2^ flasks until they reached 80% confluence and were then infected with trachyzoites of the RH strain at a multiplicity of infection of 3 parasites per host cell. 24 h after infection, BPD (D216305, Sigma Aldrich) was added at a concentration of 50 µM or not. After 48 h of growth for both treated and untreated cell lines, the infected host cells were scraped, and parasites were released by three passages through a 26 G needle. To eliminate cell debris, parasites were filtered through glass wool and then centrifuged three times in ice-cold DPBS (14190–094, Gibco). After the last wash, 1/10^th^ of the solution was kept to quantify the protein content of the parasite using a bicinchoninic acid assay (UP40840A, Interchim). For each sample, the yield was typically 2.10^8^ parasites corresponding to approximately 800 µg of total protein. The parasites were finally pelleted and rapidly frozen before being processed for lipidomic analyses.

For the quantitative analysis of neutral lipids, lipids were extracted according to Bligh and Dyer [28] in dichloromethane/methanol/water (2.5:2.5:2, v/v/v) in the presence of the internal standards (stigmasterol, cholesteryl heptadecanoate, glyceryl trinonadecanoate, and DG28). The organic phase was evaporated to dryness and dissolved in 40 µL CH_2_Cl_2_:MeOH (90:10 v/v). The SPE phase was washed with 2X1mL CH2CL2. Neutral lipids were eluted with 2 × 1 mL CH_2_Cl_2_:MeOH (90:10,v/v). Samples were dried/recovered in 2 × 80 µL ethyl acetate. Evaporation was performed and the final recovery was performed in 20 µL ethyl acetate. The lipid extract (1 µL) was analysed using a gas chromatography flame ionization detector on a GC TRACE 1600 Thermo Electron system using an RTX-5 Restek column (5% polysilarylene, 95% polydimethylsiloxane, 5 m X 0.25 mm i.d, 0.25 µm film thickness) [29]. The oven temperature was programmed to increase from 190°C to 350°C at a rate of 5°C/min, and the carrier gas was hydrogen (5 mL/min). The injector and detector were set to 315°C and 345°C, respectively.

For the quantitative analysis of phospholipids (PL), samples were extracted according to Bligh and Dyer [28] in dichloromethane/water/methanol 2% acetic acid (2.5:2:2.5, v/v/v) in the presence of 20 µL internal standards (TG19) and 40 µL internal standards (PC 13:0/13:0; Cer d18:1/12:0; PE 12:0/12:0; SM d18:1/12:0; PI 17:0/14:1; PS 12:0/12:0). After centrifugation at 2500 rpm for 6 min, the lipid extract was evaporated to dryness and dissolved in 50 µL of methanol. The PL extracts were analysed using an Agilent 1290 UPLC system coupled to a G6460 triple quadrupole mass spectrometer (Agilent Technologies) and MassHunter software for data acquisition and analysis. A Kinetex HILIC column (Phenomenex, 50 × 4.6 mm, 2.6 µm) was used for LC separation. The column temperature was maintained at 40°C. Mobile phase A was acetonitrile, and mobile phase B was 10 mM ammonium formate in water at pH 3.2. For ceramide, PE, PC, and SM, the gradient was as follows: 10–30% B for 10 min; 10–12 min, 100% B; and then back to 10% B at 13 min for 2 min prior to the next injection. The flow rate of the mobile phase was 0.3 mL/min and the injection volume was 2 µL. For PI and PS, the gradient was as follows: from 5% to 50% B for 10 min and then back to 5% B for 10.2 min for 9 min prior to the next injection. The flow rate of the mobile phase was 0.8 mL/min and the injection volume was 5 µL. Electrospray ionization was performed in the positive mode for Cer, PE, PC, and SM analysis and in the negative mode for PI and PS analysis. The needle voltage was set at 4 kV and −3.5 kV, respectively. Analyses were performed in the Selected Reaction Monitoring detection mode (SRM) using nitrogen as the collision gas. Ion optics and collision energies were optimized for each lipid class. Finally, peak detection, integration, and quantitative analysis were performed using the MassHunter Quantitative Analysis software (Agilent Technologies).

### Statistical analyses

Unpaired Student’s t-test was used for comparisons between two groups, and the Mann-Whitney non-parametric test was used for particle size comparison (cysts or lipid droplets). They were performed using Prism v8.3 (Graphpad). Unless specified otherwise, values were expressed as mean ± standard deviation (SD).

## Results

### Iron chelation impacts the growth of types I, II and III of *T. gondii*

**Figure 1.**
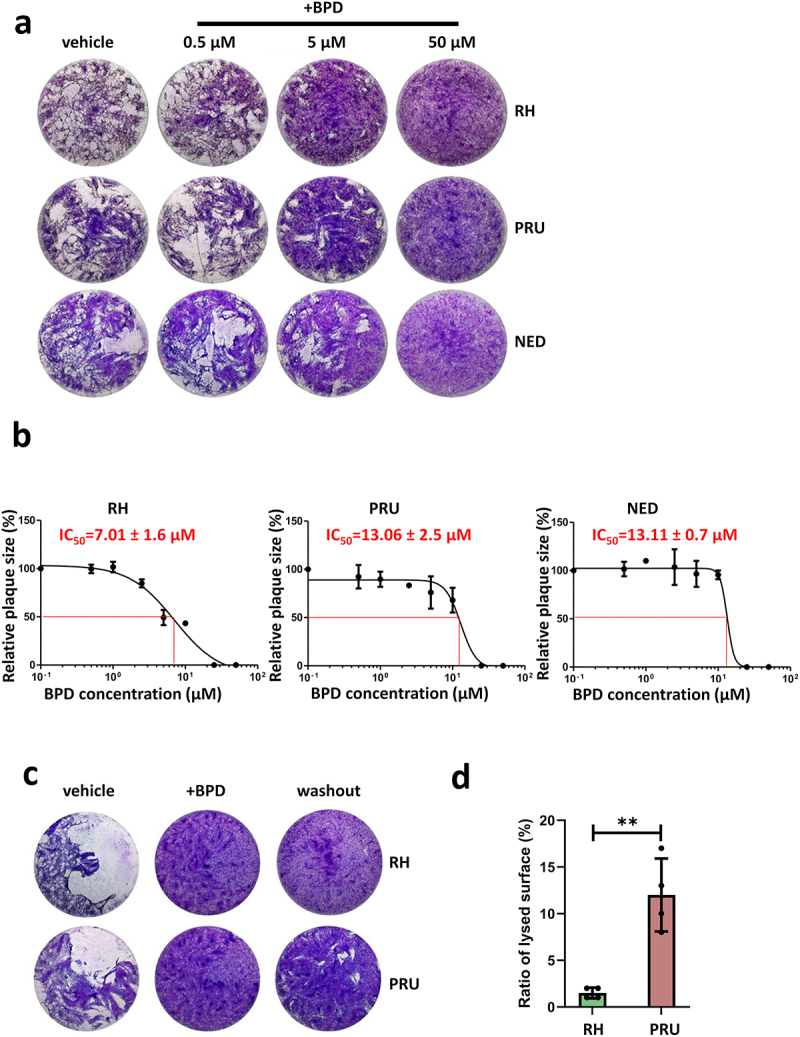
The iron chelator BPD differentially impacts growth of the type I, II and III strains of *T. gondii*. (a) Plaque assays were performed in the presence or absence of BPD: parasites were added onto HFF monolayer for 7–10 days and lysis plaques were imaged. Included was a control for which the vehicle (dimethyl sulphoxide) only was used. (b) The half maximal inhibitory concentration (IC_50_) of BPD for type I, II and III strains of *T. gondii* was calculated from plaque assays (as described in A) by assessing relative plaque size compared with the vehicle control. Data are from *n* = 3 independent experiments (except for NED, *n* = 2). Shown are mean values ± SD. (c) Parasites of the RH and PRU strains were treated for 3 days with 50 µM of BPD, and then treatment continued (+BPD) or the chelator was washed out and parasites were left to grow for another 7 days before imaging of the plaques. (d) Quantification of the relative total lysed area for the RH and the PRU parasites (compared with the vehicle control) in washout assays performed as described in C. Data are mean values ± SD from *n* = 4 independent experiments. ** denotes *p* ≤ 0.01, Student’s *t*‐test.

To assess the impact of iron chelation on the growth of *T. gondii* tachyzoites with different cystogenic capacities, we performed plaque assays on type I (RH), type II (PRU), and type III (NED) parasites in the presence of different concentrations of BPD. Plaques are formed by successive lytic cycles of the parasites (invasion/replication/egress); thus, measuring their area provides a simple and precise assessment of parasite growth. We observed a complete absence of plaques at 50 µM BPD, and an already important impact on plaque size with 5 µM BPD (Figure 1(a)). The impact at 5 µM was potentially more pronounced for the RH compared with the cystogenic PRU and NED strains, and we subsequently performed a detailed assessment of the half-maximal inhibitory concentration (IC_50_) of BPD for all strains using increasing concentrations of this compound in plaque assays. We found that the IC_50_ values of BPD for the three strains were in the same low micromolar range, although cystogenic strains, with IC_50_ values of approximately 13 µM, were slightly less affected than the RH strain, for which the IC_50_ value of BPD was approximately 7 µM (Figure 1(b)). To verify that BPD is impacting parasite growth through the chelation of iron, we performed plaque assays with BPD incubated in the presence of a supplementation of exogenous iron: for the three cell lines, this allowed at least a partial restoration of growth (Fig. S1A), which confirms that the impact of BPD on parasite fitness likely comes from the consequences of iron deprivation.

Plaque assays allow the global evaluation of parasite fitness; however, the absence of plaques does not necessarily reflect the death of the parasites, but could also be due to the parasites being still alive but unable to egress, or being largely slowed down in growth. To obtain further information on the ability of iron depletion to kill tachyzoites of the three strains, we performed a reversibility assay by incubating the parasites with a high dose of BPD (50 µM – a concentration that completely blocks plaque formation, Figure 1(a)) for three days, and then washing out the drug to allow them to potentially recover for at least a week before measuring plaque size. When performing this on either the RH strain or the cystogenic PRU strain, we noticed that while very few and very little plaques were formed by the former, the latter was able to recover to a significant extent (Figure 1(c,d)). This suggests that, although also largely impacted by BPD treatment, cystogenic strains such as PRU may be able to survive iron starvation more efficiently.

### Acute iron depletion strongly impacts the parasite ultrastructure

**Figure 2.**
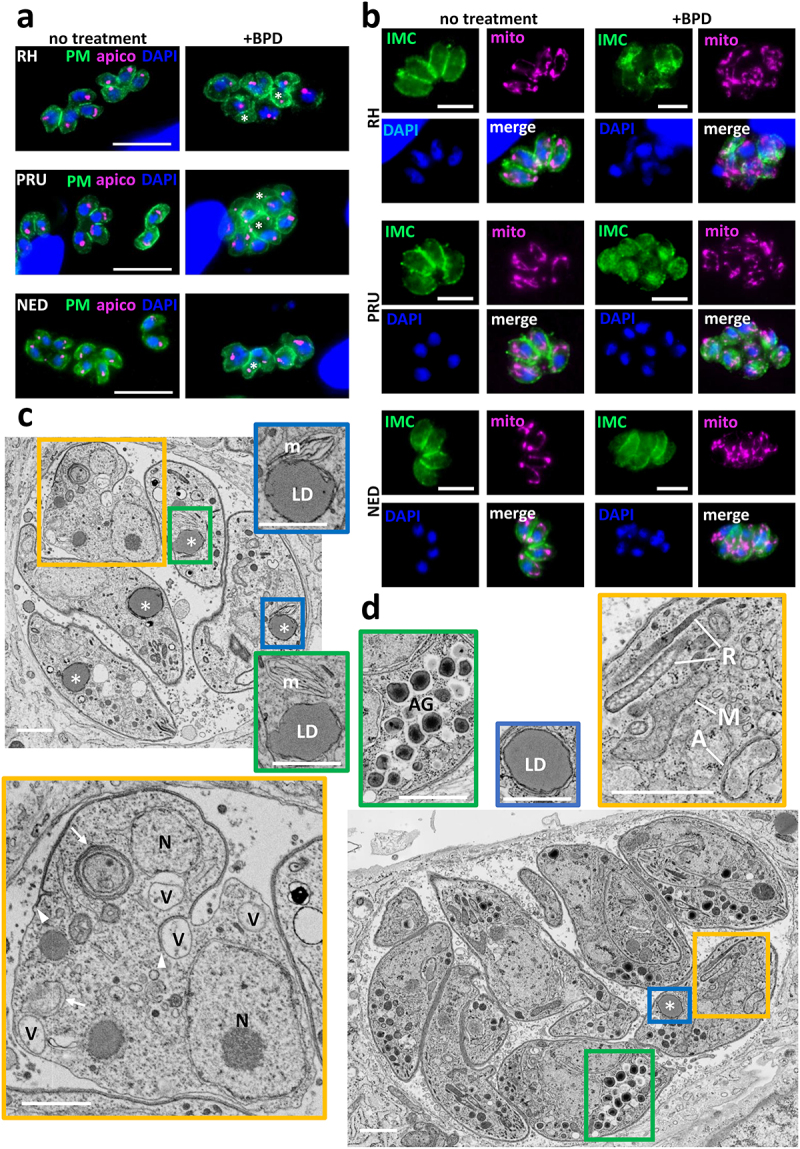
Acute iron chelation by BPD strongly impacts parasite morphology. (a) Immunofluorescence assay showing co-staining of the parasite plasma membrane (“PM,” labelled with the anti-SAG1 antibody) and the apicoplast (“apico,” labelled with the anti-PDH E2 subunit antibody) in parasites from the type I, II and III strains treated or not for 2 days with BPD. Asterisks denote parasites with either nucleus or apicoplast segregation defects. DAPI was used to stain DNA. Scale bar = 10 µm. (b) Immunofluorescence assay showing co-staining of the parasite inner membrane complex (“IMC,” labelled with the anti-IMC3 antibody) and the mitochondrion (“mito,” labelled with the anti-ATPase beta subunit antibody) in parasites from the type I, II and III strains treated or not for 2 days with BPD. DAPI was used to stain DNA. Scale bar = 5 µm. (c) Electron microscopy analysis of the effects of BPD treatment on the RH strain: asterisks denote lipid droplets (LD) which are magnified on selected insets, along with adjacent multilamellar membranes (m). The main inset shows a dividing parasite with duplicated nuclei (N) that displays interrupted inner membrane complex (arrowheads), as well as many vacuoles (V) and membranous structures potentially resembling autophagic vesicles (arrows). Scale bar = 1 µm. (d) electron microscopy analysis of the effects of BPD treatment on the PRU strain: the vacuole contains parasites dividing asynchronously, and displaying structures resembling amylopectin granules (AG, inset), the asterisk denotes a lipid droplet (LD, inset) in a parasite that shows a rather normal aspect for the mitochondrion, rhoptries secretory organelles, apicoplast (M, R and A, in the inset, respectively). Scale bar = 1 µm.

We performed immunofluorescence assays (IFAs) on type I, II, and III parasites treated for two days with 50 µM BPD to assess the effect of acute iron depletion on parasite integrity. We used markers of the pellicle of the parasite (constituted by the plasma membrane and a double-membrane complex known as the inner membrane complex (IMC) [30]) and of the mitochondrion and apicoplast, two endosymbiotic organelles that host iron-containing proteins [8,9,27]. We observed important alterations in the pellicle and problems with DNA or apicoplast segregation in daughter cells (Figure 2(a)); however, there was no major collapse or fragmentation of the endosymbiotic organelle (Figure 2(a,b)). RH parasites were generally more affected than the parasites of the type II and III strains, especially regarding the integrity of the pellicle outlining the intracellular parasites (Figure 2(a,b)).

We next used electron microscopy (EM) to assess the impact of BPD treatment on parasites at the subcellular level. Consistent with our IFA observations, we noticed that parasites of the RH strain displayed organelle segregation problems and discontinuous IMC (Figure 2(c)). In contrast to the synchronized budding and coordinated segregation of organelles that occur under normal growth conditions, BPD treatment seemed to lead to budding of daughter cells without proper incorporation of nuclear material for instance (Fig. S2). In addition, we observed vacuolization and accumulation of membranes, which in some instances surrounded cytosolic material, similar to autophagic vesicles [31]. Finally, we observed the frequent presence of large structures resembling lipid droplets (LDs) in the parasites (Figure 2(c)). EM analysis of the effect of BPD on the cystogenic type II PRU strain showed budding problems in some dividing parasites, but there was an overall better preservation of the internal membranes and organelles (Figure 2(d)). BPD-treated PRU parasites displayed LDs similar to those found in the RH strain, but in addition, we observed structures resembling granules of amylopectin, a storage polysaccharide often found in bradyzoites [32], suggesting that they might initiate stage conversion (Figure 2(d)).

Overall, our data show that acute treatment with the iron chelator BPD has a strong effect on the growth and morphology of *T. gondii* tachyzoites, yet it might have a more detrimental impact on non-cystogenic strains compared to cystogenic strains.

### Iron starvation of cystogenic strains leads to an efficient conversion into bradyzoites

**Figure 3.**
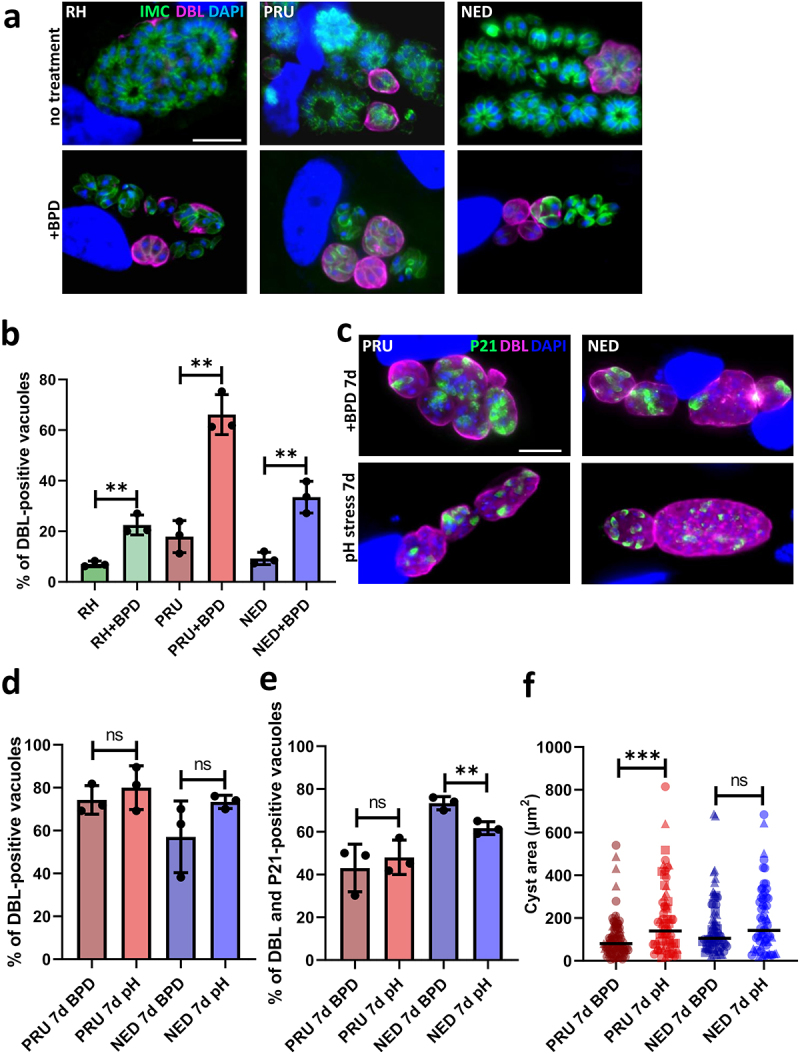
Iron deprivation by BPD triggers stage conversion into bradyzoites. (a) Parasites from the type I, II and III strains treated or not for 2 days with BPD were stained for the inner membrane complex (IMC) to outline the parasite shape and co-stained with *dolichos biflorus* lectin (DBL) to detect the maturation of the parasitophorous vacuole membrane into a cyst wall. DAPI was used to stain DNA. Scale bar = 10 µm. (b) quantification of DBL-positive vacuoles after 2 days of BPD treatment. Data are mean values ± SD from *n* = 3 independent experiments. At least 50 vacuoles were counted in each experimental condition. ** denotes *p* ≤ 0.01, Student’s *t*‐test. (c) type II and III cystogenic strains were kept for 7 days in the presence of BPD or stage conversion was induced by alkaline pH stress for the same duration and co-staining was performed for the cysts wall (DBL) and the late bradyzoite marker P21. (d) quantification of DBL-positive vacuoles after 7 days of BPD treatment or alkaline pH stress. Data are mean values ± SD from *n* = 3 independent experiments. At least 30 vacuoles were counted in each experimental condition. ns: not statistically significant, Student’s *t*‐test. (e) quantification of DBL-labelled vacuoles containing P21-positive parasites after 7 days of BPD treatment or alkaline pH stress. Data are mean values ± SD from *n* = 3 independent experiments. at least 20 vacuoles were counted in each experimental condition. ** denotes *p* ≤ 0.01, ns: not statistically significant, Student’s *t*‐test. F. measurement of cyst area after 7 days of BPD treatment or alkaline pH stress. Data are mean values ± SD from *n* = 3 independent experiments. At least 25 DBL-positive cysts/vacuoles were measured in each experimental condition. *** denotes *p* ≤ 0.001, ns: not statistically significant, Student’s *t*‐test.

As a number of stresses, including nutrient starvation, can initiate the conversion of tachyzoites into bradyzoites, and because of our observation that cystogenic strains such as PRU better survive iron depletion (Figure 1(c,d)), we next sought to quantitatively assess the ability of BPD to initiate differentiation. The lectin from the plant *Dolichos biflorus* (DBL) specifically recognizes a cyst wall glycoprotein called CST1, which is synthesized early upon initiation of stage conversion and accumulates as differentiation progresses [33]. Imaging with fluorescently labelled DBL allowed the identification of the characteristic peripheral labelling of vacuoles that initiated cystogenesis after short-term BPD treatment (Figure 3(a)). This staining can already be found to occur to some extent under normal *in vitro* culture conditions with cystogenic strains, which usually display some degree of spontaneous differentiation (Figure 3(a,b)). However, after two days of BPD treatment, we observed an increased proportion of vacuoles displaying DBL staining (Figure 3(a,b)), although this was more pronounced in the cystogenic type II and III strains than in the type I strain (65%, 33%, and 22% of DBL-positive vacuoles, respectively).

As the BPD washout experiment suggested that cystogenic strains are potentially able to cope better with iron depletion (Figure 2(a,b)) and as it may be due to their potential to convert into bradyzoites, we performed long-term incubation with BPD on the PRU and NED strains to assess their ability to convert to more mature cysts. Conversion into bradyzoites is a continuum that occurs over the course of several days, and while DBL staining is an early marker of cystogenesis the use of antibodies against P21, a late marker of bradyzoite differentiation whose expression can be detected after 7 days of alkaline pH-induced differentiation [34,35], is more suitable for the labelling of mature cysts. We performed alkaline pH stress (the most common way to induce conversion to bradyzoites *in vitro* [36]) on the PRU and NED strains and, in parallel, BPD treatment for up to 7 days, and assessed stage conversion by DBL staining and bradyzoite maturation by P21 staining. We observed that both alkaline pH stress and BPD treatment led to comparable conversion rates as assessed by DBL staining (Figure 3(d)), and that the proportion of DBL-positive vacuoles that contained P21-positive mature bradyzoites was also similar (Figure 3(c,e)). However, the cysts generated upon BPD treatment were slightly smaller than those generated through alkaline pH stress (especially for the PRU strain, Figure 3(f)), suggesting that iron chelation has some impact on bradyzoite growth.

Thus, our data confirm that iron chelation through BPD treatment leads to efficient conversion into mature bradyzoites for the cystogenic strains of *T. gondii*.

### BPD causes a marked perturbation of lipid homeostasis both in host and parasite cells.

**Figure 4.**
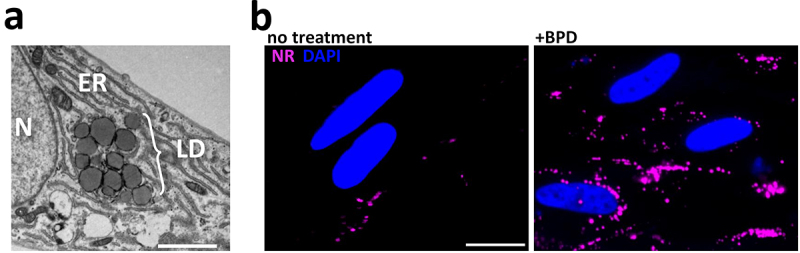
Iron chelation by BPD leads to lipid droplet accumulation in uninfected host cells. (a) Representative transmission electron microscopy micrograph showing the accumulation of lipid droplets (LD) in a fibroblast after 2 days of BPD treatment. ER: endoplasmic reticulum, N: nucleus. Scale bar = 2 µm. (b) fluorescence microscopy picture of fibroblasts either untreated (left) or treated (right) with BPD for 2 days showing that iron chelation leads to an increase in LD, which were labelled with Nile red (NR). DNA was stained with DAPI. Scale bar = 20 µm.

**Figure 5.**
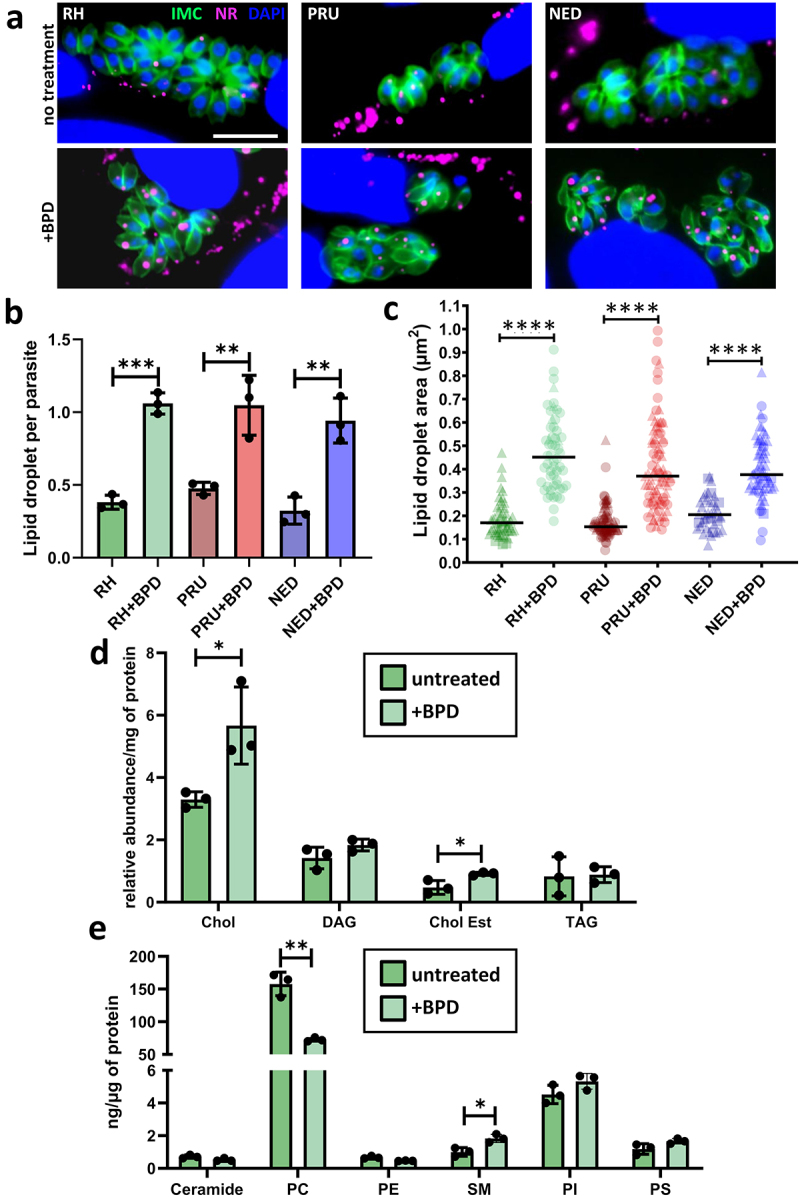
Treatment by BPD affects lipid homoeostasis in *T. gondii*. (a) Intracellular parasites from the type I, II and III strains were treated or not for 2 days with BPD and stained with nile red (NR) to label the lipid droplets (LD) and counterstained for the inner membrane complex (IMC) protein IMC3 to outline the parasite shape. DNA was stained with DAPI. Scale bar = 10 µm. (b) Quantification of LD numbers per parasite after treatment or not with BPD for 2 days. Data are mean values ± SD from *n* = 3 independent experiments. At least 280 parasites were counted in each experimental condition. ** denotes *p* ≤ 0.01, *** denotes *p* ≤ 0.001, Student’s *t*‐test. (c) measurement of LD area in parasites after treatment or not with BPD for 2 days. Data are mean values from *n* = 3 independent experiments. At least 40 LDs were measured in each experimental condition. Symbols are matched between identical experimental groups. **** *p* ≤ 0.0001, non-parametric Mann-Whitney test. (d) analysis of neutral lipid content in parasites after treatment or not with BPD for 2 days. Chol: cholesterol, DAG: diacylglycerol, Chol Est: cholesteryl esters, TAG: triacylglycerol. Data are mean values ± SD from *n* = 3 independent experiments. * denotes *p* ≤ 0.05, Student’s *t*‐test. (e) analysis of phospholipid content in parasites after treatment or not with BPD for 2 days. PC: phosphatidylcholine, PE: phosphatidylethanolamine, SM: sphingomyelin, PI: phosphatidylinositol, PS: phosphatidylserine. Data are mean values ± SD from *n* = 3 independent experiments. * denotes *p* ≤ 0.05, ** denotes *p* ≤ 0.01, Student’s *t*‐test.

When observing by EM the impact at a sub-cellular level of BPD treatment, we noticed an accumulation of LD in intracellular parasites (Figure 2(c)). The induction of LD was not restricted to the parasites, as they were also seen in the host cells: in fact, even in uninfected host fibroblasts, short-term (2 days) treatment with BPD led to a marked increase in LD, as seen both by EM and by fluorescence microscopy using Nile Red to stain the LDs (Figure 4(a,b)). This is in line with recent findings obtained using several different iron chelators that were shown to induce quick and robust LD formation in mammalian cells [37–39]. We also observed a strong increase in LDs within the parasites of all three strains (Figure 5(a)), for which incubation with BPD induced both an increase in LD number (Figure 5(b)) and size (Figure 5(c)). Like for parasite growth, this effect was reversed by the supplementation of iron (Fig. S1B), suggesting that the impact on LD biogenesis is directly linked to iron deprivation.

Homeostasis of host lipids has important consequences on the parasites’ own lipid metabolism, as tachyzoites are able to scavenge lipids stored in host LDs and can incorporate them into their own membranes and LDs [40]. Thus, we sought to determine the lipidomic profile of RH parasites treated for two days with BPD. LDs are typically composed of a central core of neutral lipids (mostly triacylglycerols and cholesterol esters) surrounded by a monolayer of phospholipids (PLs). The analysis of the parasite’s neutral lipids revealed a marked increase for cholesterol as well as for its fatty acid esters (cholesterol esters), which are typical components of LDs [41] (Figure 5(d)). Analysis of the parasite’s PL content highlighted two significant changes upon BPD treatment: a decrease in phosphatidylcholine and an increase in sphingomyelin (Figure 5(e)). Interestingly, phosphatidylcholine is one of the major PLs coating the surface of LDs, and it has been shown previously that a decrease in phosphatidylcholine leads to an increase in LD size [42] (when the levels of phosphatidylcholine decrease, LDs coalesce via fusion to minimize the surface of the interface and to optimize surface coverage by the PL), which may contribute to the enlargement of LDs observed in addition to their increased numbers after BPD treatment. An increase in sphingomyelin may be attributed to either an increase in scavenging from the host [43] or an increase in the activity of the parasite’s own sphingomyelin synthase [44], an enzyme that may generate sphingomyelin or neutral lipids from phosphatidylcholine, which may then be incorporated into LDs [45].

The metabolism of long-term persisting bradyzoites relies on the storage of nutrients, such as carbohydrates [46], as well as potentially lipids [47]. Thus, we assessed whether the marked accumulation of LDs upon iron chelation was due to an indirect effect linked to parasite differentiation. To this end, we assessed LD size and quantification in type II and III bradyzoites obtained after short- (3 days) and long-term (7 days) differentiation under alkaline pH stress conditions (Fig. S3A). We observed an increase in LD size in converting parasites, especially for the type II strain, and in late-differentiated parasites (Fig. S3C), in accordance with a previous study showing that differentiated bradyzoites contain large LDs [47]. However, the number of LDs per parasite did not change (Fig. S3B), in sharp contrast to the increase in LDs number induced by BPD treatment (Figure 5b, Fig. S3A). Moreover, although the type I strain is not prone to cystogenesis, it displayed LDs increasing in size and numbers in similar proportions to those of the cystogenic strains when treated with BPD. Altogether, this suggests that the increase in LDs upon BPD treatment is likely not due to conversion into bradyzoites, but rather to the more general perturbation of lipid homoeostasis caused by iron chelation.

## Discussion

The ability of *T. gondii*‘s developmental stages to ensure nutrient supply for optimal proliferation and persistence is crucial to the virulence of the parasite. Although metabolic requirements of reference strains, which have been maintained during successive passages in animals or cell culture, may differ from non-laboratory-adapted strains [48,49], they have been instrumental in defining currently known metabolic interactions between *T. gondii* and its host cells [50]. Nutrient limitation, particularly iron restriction, is a powerful innate immune defence mechanism for host cells to control microbial pathogens [51]. This competition for cellular iron is a delicate balance between the host and microbial systems, which is at the heart of the concept of nutritional immunity [52], with complex implications and consequences that can also be potentially harmful to the host cells themselves. Our results show a pronounced impact of acute iron depletion on the parasites, with perturbation of membrane homoeostasis and defaults in the segregation of organelles and DNA in developing tachyzoites. This is not surprising as the two major classes of cofactors that utilize iron in cells, the iron-sulphur cluster and haem, are central to proteins that are typically involved in key cellular functions such as DNA replication and repair, transcriptional and translational control, respiration, and cellular detoxification processes [53]. Not only does limiting access to iron seem to be part of the immune response to *T. gondii* infection [12], but drug-based iron chelation has also been suggested as a potential strategy against the parasite [11]. The impact of iron depletion on *T. gondii* has thus been gaining momentum recently, with studies investigating, for instance, the global consequences of iron chelation on the transcriptome of the parasite [54,55]. The main and most obvious trend in the transcriptional profile of iron-depleted *T. gondii* tachyzoites is the induction of bradyzoite-specific genes [54,55]. Our own quantitative data demonstrate an initiation of stage conversion in all parasite strains we tested, including type I parasites; however, we have shown that there is a particularly robust induction of differentiation in type II and III cystogenic strains. It is known that reduced availability of nutrients, along with withdrawal from cell cycle progression or proinflammatory responses, for example, can induce tachyzoite-to-bradyzoite differentiation and thus cyst formation [56]. We now show that the use of iron chelators, despite having detrimental effects on some parasites, is another tool that can be considered to help generate long-term *in vitro* bradyzoites in cystogenic strains, either alone or in combination with other stress-inducing factors already available [36].

*T. gondii* is an intracellular parasite that is perfectly adapted to thrive in its host cell, using a combination of *de novo* synthesis and scavenging from the host to obtain essential metabolites [49,50]. Drug-based approaches that interfere with the supply of key elements, such as iron, affect both the host and parasites, and it may be complicated to disentangle the direct indirect effects on the parasites. While recent investigations at the transcriptomic level of the impact of iron chelation on *T. gondii* tachyzoites did not highlight a particular metabolic pathway that could potentially be affected [54,55], our morphological analysis of BPD-treated parasites showed a pronounced effect on host and parasite lipid homoeostasis, as illustrated by the strong accumulation of LDs. *T. gondii* infection results in the recruitment of host LDs and increases their biogenesis through modification of the host neutral lipid metabolism [57,58]. Recent converging evidence has shown a strong potential for iron chelators to induce LD formation in mammalian cells [37–39], which we confirmed in this study. In mammalian cells, it has been suggested that iron depletion induces fatty acid oxidation and leads to mitochondrial dysfunction [39], but the precise molecular mechanisms involved remain unknown. Our lipidomic data suggest that the consequences of iron depletion on host cell lipids subsequently impact the parasite’s lipid homoeostasis through an increased influx of cholesterol. It is also possible that there is a more direct effect of the chelator on the parasites that would stimulate LD synthesis in a fashion similar to what happens in the mammalian host cell. Additional investigation, using short term iron chelation and a combination of targeted transcriptomics, proteomics and metabolomics analyses may allow the identification of parasite-specific factors.

The biogenesis of LDs is part of an integrated stress response to cellular injuries, and not only is their biogenesis quite logically induced in cells exposed to excess amounts of lipids, but also in conditions of acute nutrient deprivation and after oxidative stress [59]. LDs contribute to the protection of cellular integrity by buffering the excess of potentially toxic lipids, maintaining energy and redox balance and preserving membrane homoeostasis. As confirmed by our microscopic observations, acute iron depletion had a pronounced impact on the parasite’s membrane homoeostasis, and the strong increase in LD formation in the parasite may not only be a consequence of the increased amount of lipids in the host, but also as a part of the parasite’s own stress response mechanism. *T. gondii* tachyzoites mitigate stress damage through an integrative stress response pathway that can, for example, be activated under conditions of nutrient starvation [60] or acute oxidative stress [61], and leads to stage conversion into the persistent bradyzoite form [62]. Conversion from fast-replicating tachyzoites to this cyst-confined developmental form is induced by a variety of stresses and, thanks to very slow replication and reduced metabolic activity, extends survival in adverse conditions [36]. Our results demonstrate that while acute iron depletion strongly and rapidly impacts the viability of type I parasites, it leads to a relatively efficient conversion into the bradyzoite form for type II and III cystogenic strains. Long-term acute iron deprivation may also be detrimental to bradyzoites, but such a treatment will likely also be damaging to host cells. In the mammalian host, bradyzoites persist in specific tissues, including the brain, where there is an overall low amount of this element [13] (although there are probably regional iron concentration differences [63]). Thus, it is possible that these forms are better adapted to respond to low iron availability. Bradyzoites are largely resistant to current therapies used against acute toxoplasmosis. Our results show the ability of cystogenic strains to efficiently convert into bradyzoites upon treatment with an iron chelator and to persist in these conditions for several days *in vitro*. Thus, regarding the use of iron deprivation as a treatment for toxoplasmosis [11], our findings are a cautionary note that it might not be particularly efficient in the context of chronic toxoplasmosis.

## Supplementary Material

Supplemental Material

## Data Availability

The authors confirm that the data supporting the findings of this study are available in the article and its supplementary materials.
