# Supplementary material for: Iron depletion has different consequences on the growth and survival of Toxoplasma gondii strains
Source: Virulence. 2024 Mar 20;15(1):2329566. doi: 10.1080/21505594.2024.2329566 (PMC10962585; doi:10.1080/21505594.2024.2329566)
Supplement: Supplemental Material [file KVIR_A_2329566_SM1589.zip › Updated supplementary files.pdf]

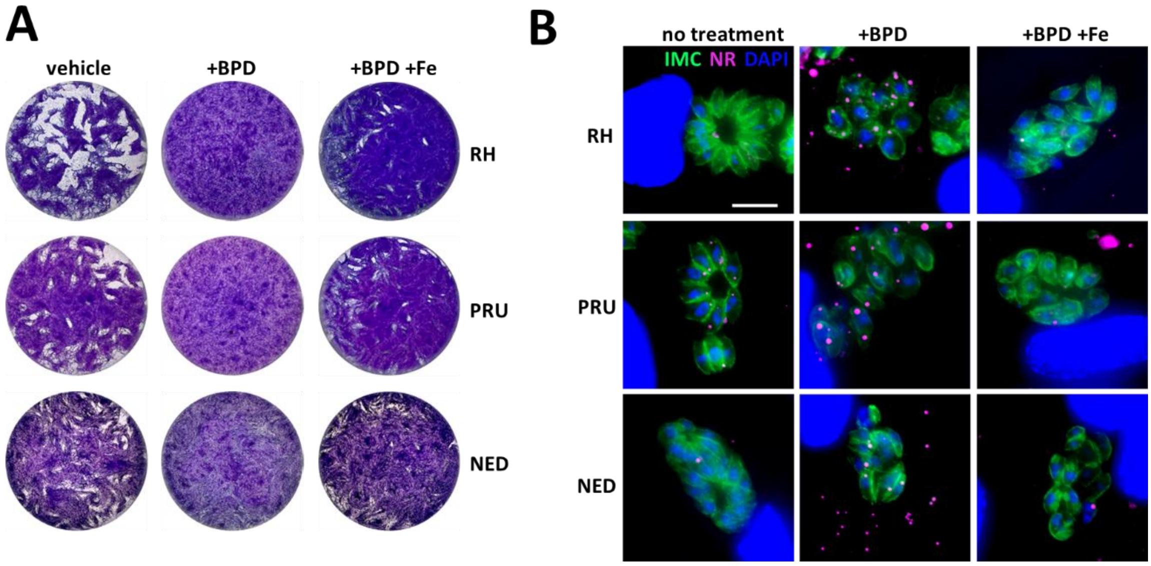

Figure S1. The addition of exogenous iron compensates for parasite growth defects and prevents lipid droplet accumulation induced by BPD. **A.** Plaque assays performed with the RH, PRU or NED strains in the presence or absence of BPD and/or exogenous iron using 100 µM $FeCl_3$: parasites were added onto HFF monolayer for 7-10 days and lysis plaques were imaged. Shown is one representative experiment out of two independent biological replicates. **B.** Representative fluorescence microscopy pictures of fibroblasts either untreated, or treated with BPD alone, or together with 100 µM $FeCl_3$ for 2 days, showing that lipid droplets, which were labelled with Nile red (NR), are not induced in the presence of additional iron. Parasites were counterstained for the inner membrane complex (IMC) protein IMC3 to outline their shape. DNA was stained with DAPI. Scale bar = 5 µm.

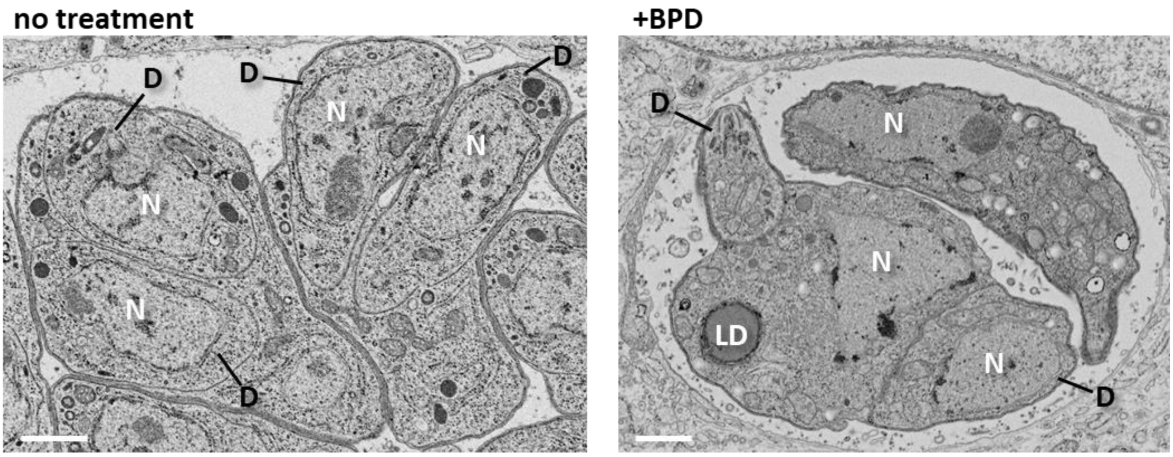

Figure S2. BPD treatment impairs daughter cell budding. The left electron microscopy image shows untreated RH parasites dividing almost synchronously, whith each daughter cell (D) having incorporated organelles like the nucleus (N). The left electron microscopy picture shows RH parasites after two days of BPD treatment, displaying asynchronous division and daughter cell budding leaving out organellar material. LD: lipid droplet. Scale bar = 1 µm.

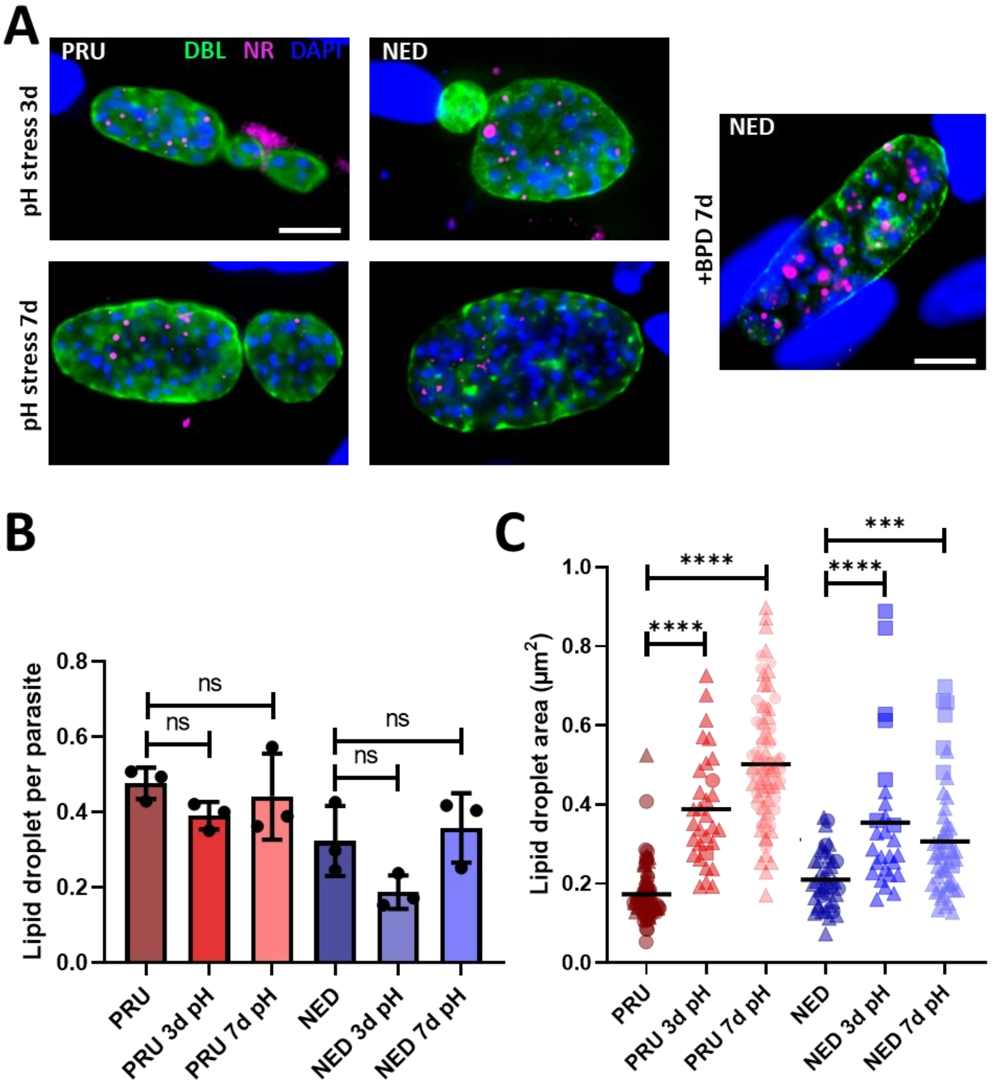

Figure S3. Lipid droplets in alkaline pH-induced cysts. **A.** Parasites of the type II and III cystogenic strains were submitted to alkaline pH-induced differentiation for 3 or 7 days and imaged for the cyst wall (DBL) and for lipid droplet (LD) content with Nile red (NR). Representative image of a cyst with type III parasites obtained after 7 days of BPD treatment is shown on the right for comparison. DNA was stained with DAPI. Scale bars = 10 µm. **B.** Quantification of LD numbers per parasite after inducing or not conversion into bradyzoites by pH stress for 3 or 7 days. Data are mean values ± SD from n = 3 independent experiments. At least 280 parasites were counted in each experimental condition. ns: not statistically significant, Student's t-test. **C.** Measurement of LD area in parasites after inducing or not conversion into bradyzoites by pH stress for 3 or 7 days. Data are mean values from n = 3 independent experiments. At least 40 LDs were measured in each experimental condition. Symbols are matched between identical experimental groups. *** $p \leq 0.001$, **** $p \leq 0.0001$, non-parametric Mann-Whitney test.
